# High-dose short-term osimertinib treatment is effective in patient-derived metastatic colorectal cancer organoids

**DOI:** 10.1038/s44276-024-00042-0

**Published:** 2024-04-03

**Authors:** Kirti K. Iyer, Dennis Poel, Anne Miggelenbrink, Wouter Kerkhof, Jorien Janssen, Lotte Bakkerus, Loek de Jong, Erik van den Hombergh, Iris D. Nagtegaal, Daniele V. F. Tauriello, Nielka P. van Erp, Henk M. W. Verheul

**Affiliations:** 1 Department of Medical Oncology, Research Institute for Medical Innovation, Radboud University Medical Centre, Nijmegen, The Netherlands; 2 Department of Medical Biosciences, Research Institute for Medical Innovation, Radboud University Medical Centre, Nijmegen, The Netherlands; 3 Department of Pharmacy, Research Institute for Medical Innovation, Radboud University Medical Centre, Nijmegen, The Netherlands; 4 Department of Pathology, Research Institute for Medical Innovation, Radboud University Medical Centre, Nijmegen, The Netherlands; 5 Department of Medical Oncology, Erasmus Medical Centre, Rotterdam, The Netherlands

## Abstract

**Background:**

Most tyrosine kinase inhibitors (TKIs) have failed in clinical trials for metastatic colorectal cancer (mCRC). To leverage the additional lower-affinity targets that most TKIs have, high-dose regimens that trigger efficacy are explored. Here, we studied unprecedented drug exposure–response relationships in vitro using mCRC patient-derived tumour organoids (PDTOs).

**Methods:**

We investigated the cytotoxic anti-tumour effect of high-dose, short-term (HDST) TKI treatment on 5 PDTOs. Sunitinib, cediranib and osimertinib were selected based on favourable physicochemical and pharmacokinetic properties. Intra-tumoroid TKI concentrations were measured using a clinically validated LC/MS-MS method. Cell death was determined using an enzyme activity assay, immunofluorescent staining and western blotting.

**Results:**

Most PDTOs tested were sensitive to sunitinib and cediranib, but all to osimertinib. Furthermore, HDST osimertinib treatment effectively blocks organoid growth. This treatment led to markedly elevated intra-tumoroid TKI concentrations, which correlated with PDTO sensitivity. Mechanistically, HDST osimertinib treatment induced apoptosis in treated PDTOs.

**Conclusion:**

Our work provides a better understanding of TKI exposure vs response and can be used to determine patient-specific sensitivity. Additionally, these results may guide both mechanistic elucidation in organotypic translational models and the translation of target drug exposure to clinical dosing strategies. Moreover, HDST osimertinib treatment warrants clinical exploration for mCRC.

## Introduction

Colorectal cancer (CRC) is responsible for causing more than 1.85 million cases worldwide and nearly 900,000 deaths every year [1]. It is a highly heterogeneous disease encompassing different genetic mutations [2], including, in the *KRAS* gene which occurs in almost half of the patients and further leads to a dismal prognosis [3–5]. Recent advances in treatment strategies—surgery, radiotherapy, chemotherapy, immunotherapy and targeted therapy—combined with improvement in early diagnosis, have increased the overall survival (OS) of patients with CRC [6]. However, the prognosis for patients with metastatic disease (mCRC) remains discouraging, with a 5-year survival rate of ~14% [7], highlighting an urgent need for novel and effective treatment strategies for these patients. Especially for those patients with *KRAS*-mutant tumours who have very limited targeted options available [8].

Increasing knowledge on the molecular basis of oncogenic pathways has helped to identify a key role of aberrant kinase signalling in mCRC development. Specific molecular alterations, including mutations, gene amplifications and translocations in protein kinases enable these pathways to be constitutively active, driving cancer cells to survive, proliferate, and metastasise. To inhibit the activity of these key enzymes, many small molecule tyrosine kinase inhibitors (TKIs) have been developed and investigated in mCRC [9–11]. Over 40 Food and Drug Administration (FDA) approved TKIs have shown promising anti-cancer activity in preclinical CRC studies. Yet, despite several clinical trials, only regorafenib has been FDA approved as monotherapy for the treatment of mCRC [12]. This huge translational failure could be attributed to the lack of models mimicking complex tumour behaviour, leading to overestimation of preclinical drug efficacy, or to the neglect of the drug exposure levels that are required for anti-cancer efficacy that are not reached in the clinic by using standard dosing. Indeed, little is known about effective intracellular concentrations in both clinical as well as in preclinical evaluations. There is also a relative lack of knowledge on tumoral pharmacology in preclinical models.

This is especially relevant for TKIs; while some are considered to be selective, many have an expanded kinase inhibitory potency at higher concentrations [13]. Multiple high-dose treatment regimens have been proposed to explore whether this concept can be leveraged to improve the efficacy of TKIs, while maintaining acceptable toxicity [14]. For instance, when sunitinib was administered as intermittent, high-dose (700 mg once every 2 weeks instead of 50 mg daily) in a phase I clinical trial, we found that this high-dose schedule was feasible and safe for heavily pre-treated patients with solid tumours [15]. Moreover, we observed promising preliminary anti-tumour effects for the high-dose schedule at which much higher concentrations were reached in the plasma and, more importantly, in tumour samples. Remarkably, a positive correlation between intra-tumoral sunitinib concentrations, measured in on-treatment biopsies, and OS was found [16], supporting the hypothesis that a concentration-dependent expansion of the kinase inhibitory spectrum may boost drug activity.

Aiming to understand these clinical observations of sunitinib in patients with advanced cancer and to identify new potential treatment options for mCRC, we now focus on TKIs that are likely to reach high plasma concentrations as well as, presumably, higher tumour concentrations [14]. To assess these TKIs’ potential for clinical translation and to be able to determine optimal drug exposure, as well as dissect the mechanism of action—we used 3D matrix-embedded tumour organoids derived from patients with mCRC by biopsies and developed methods to determine intracellular drug concentrations. The aim of this study is to determine drug sensitivity of patient-derived tumour organoids (PDTOs) in conjunction with the effective drug concentrations required for anti-tumour activity of TKIs upon HDST exposure.

## Materials and methods

### Human tissues

Liver metastasis specimens from mCRC patients were acquired from 2 clinical trials (SUNRISE-CRC, NCT03909724) and ORCHESTRA, NCT01792934). These studies and subsequent collection of tumour materials were approved by the medical ethics committee of the VUmc and were conducted in accordance with Good Clinical Practice and the Declaration of Helsinki. All participants provided written informed consent.

### Patient-derived tumour organoid culture

PDTOs were established from both needle biopsies and resection material as previously described [17]. Detailed procedure for establishing PDTOs is described in the supplementary material. In all experiments, PDTOs were disaggregated into single cells to control for equal numbers between conditions. PDTOs were allowed to reform (into organoid structures) for 5 days before any treatment/analysis.

### TSO500 panel for genetic characterisation of PDTOs

To determine mutations and copy number variations in the PDTOs, the TruSight Oncology 500 (TSO500) assay (Illumina) was used. The DNA of fresh collected PDTOs was isolated using the DNeasy Blood and Tissue Kit (Qiagen, #69504). For library preparation, 60 ng was used as input and performed using the hybrid capture-based TruSight Oncology 500 Library Preparation Kit (Illumina) following the manufacturer’s protocol. Sequencing was performed on a NextSeq 500 system (Illumina) with 10 libraries sequenced per run (NextSeq high-output). For all 5 PDTOs the median unique coverage of all exonic regions was >500×. The raw sequencing data was processed and analysed by the TruSight oncology 500 Local App V.2.0 (Illumina) followed by an in-house developed pipeline as previously described [18]. Mutations and copy number variations in the mCRC PDTOs were identified by bioinformatical approaches and manually curated. Pathogenicity of variants was determined based on, various knowledgebases (ClinVar, OncoKB, JAX CKB, MyCancer Genome, and COSMIC) and literature.

### Immunohistochemistry (IHC) staining

To check whether the PDTOs matched the patient tumour phenotypically and to confirm their CRC origins, haematoxylin and eosin (H&E) and CRC specific staining were performed. Detailed protocol is described in the supplementary material.

### Selection of TKIs

The selection of the multikinase inhibitors for this study was based on the requirement of dose-proportional pharmacokinetics over a wide dosing range—which makes alternative dosing schedules feasible in the clinic—focusing on FDA- and/or European Medicines Agency (EMA)-registered drugs primarily. Further, the TKIs were selected based on: 1) an octanol–water partition coefficient (log P) that should not exceed >5 to ensure optimal membrane passage; 2) pKA strongest acid >2⋅5 and strongest basic <11, because this will lead to optimal absorption; 3) excellent solubility (BSC class 1 or 3); 4) high bioavailability (or the option to enhance this); and 5) the potency to inhibit multiple “off” target kinases with increased exposure. Also see reference [14]. This led to the initial selection of sunitinib (Lc laboratories, S-8877); cediranib (TargetMol, T2500) and osimertinib (Medchem, HY-15772). A highly selective TKI, imatinib (Lc laboratories, I-5577) with similar chemical and pharmacokinetic properties was also selected (Supplementary Table 2).

### Exposure-response analysis

PDTOs were treated with different concentrations of the TKIs, ranging from 0.625 µM to 20 µM. The growth rate was determined after 7 days of drug exposure (day 12) and compared with the t_0_ measurements. Detailed treatment protocol is described in the supplementary material.

### High-dose, short-term exposure of TKIs

PDTOs were subjected to high-doses of TKIs for various time intervals. After each time interval, the drug containing medium was removed and the wells were washed with HBSS (Lonza, 14175095) and replaced with fresh +14 culture medium. After wash-out, the PDTOs containing the +14 culture medium were allowed to recover until 7 days after treatment with the TKIs. On day 12, the readout was performed by CTG assay. Detailed treatment conditions are described in the supplementary material.

### Determining intra-tumoroid TKI concentrations

PDTOs were treated with 20 µM HDST TKIs for 1, 3 and 6 h. These time points arose from previous in vitro work [19] and aim to mimic the peak in drug levels that occur in patients just after intermittent high dosing. Exposure times >6 h were problematic due to initiating cell lysis. Culture medium (before and after treatment) was collected for each condition. The PDTOs were collected in Cell Recovery Solution (Corning, 354253) according to the manufacturer’s protocol. The resulting dry pellet was weighed. The collected media and the tumoroid samples were analysed to quantify the TKIs. For quantification, a validated liquid chromatography-tandem mass spectrometry (LC-MS/MS) assays were used. The LC-MS/MS system consist of an Acquity UPLC® H-class combined with a TQ-S micro detector (Waters®, Milford, USA) with MassLynx software. Detailed information on sample collection for mass balance validation and further quantification is described as supplementary material.

### Caspase-Glo® 3/7 3D assay

To detect cell death after HDST TKI treatment, Caspase-Glo® 3/7 reagent (Promega, G8981) was used according to manufacturer’s protocol and the luminescence was measured using the Victor3^TM^ plate reader. Further details are described in the supplementary material.

### Immunofluorescent (IF) staining with Cleaved caspase-3 antibody

PDTOs subjected to HDST TKI treatment were fixed and embedded in paraffin blocks as described previously. These were cut in slides, which were stained with cleaved caspase-3 antibody and DAPI according to manufacturer’s protocol. LSM900 confocal microscope (Zeiss) was used to visualise the staining. Detailed protocol described in supplementary material.

### Western blot

HDST TKI treated PDTOs were lysed using a 10 X Cell lysis buffer (Cell signalling technology, 9803 S) supplemented with phosphatase (Roche, 4906837001) and protease inhibitors (Roche, 11697498001). The lysates were mixed with 2 X Laemelli sample buffer (Biorad, 1610737), supplemented with β-mercaptoethanol (Gibco, 31350010) boiled for 6 min at 96 °C and further resolved on a 10% running gel followed by transfer to polyvinylidene difluoride membrane (Millipore). The membranes were blocked with 5% non-fat milk in wash buffer (TBS + 0.1% Tween-20). Next, immunoblotting was performed with primary antibodies at 4 °C overnight. Next day, the blot was incubated with secondary antibodies. Blots were analysed using the chemiluminescence method (LAS4000) and were developed using Super Signal West Femto (Thermo Fisher Scientific, 34094) reagent. Detailed protocol described in supplementary materials.

### Statistical analysis

All quantitative data are presented as means ± SEM. Statistical significance was determined using GraphPad Prism software (version 9.4.1). The statistical significance between groups was analysed using nonparametric Kruskal–Wallis test. Multiple groups were analysed using two-way analysis of variance (ANOVA) with Sidak’s multiple comparisons test. *P*-values of <0.05 were considered statistically significant.

## Results

### PDTOs from mCRC retain patient-specific characteristics

**Fig. 1:**
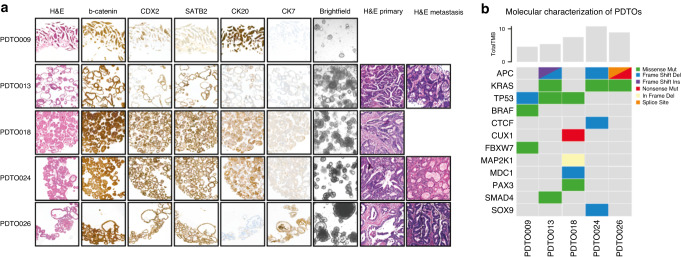
Histopathological and genotypic characterisation of established mCRC PDTOs. **A** Histopathological comparison of patient-derived tumour organoids (PDTOs) with metastatic source material and matched primary tumours (where available) by haematoxylin & eosin (H&E) staining, and immunohistochemical staining of established mCRC PDTOs with CRC specific markers (β-catenin, CDX2, SATB2 and CK20) and negative marker (CK7). **B** Overview of the genotypic characterisation of the PDTOs. TMB tumour mutational burden (average mutations per mega base). Colours indicate the type of mutation.

We established a biobank of PDTOs from CRC metastases. Of the five microsatellite-stable (MSS) liver metastasis-derived PDTOs used in this study, four (PDTO009, PDTO013, PDTO018 and PDTO026) were established from needle biopsies and one (PDTO024) was established from resection material (Supplementary Table S1). The PDTOs displayed a distinct range of histopathological features that were comparable to the patients’ tumours (Fig. 1A).

Immunohistochemistry (IHC) staining with CRC-specific markers confirmed the CRC origin of the established PDTOs (Fig. 1A). Although CRC is usually CK7 negative, PDTO026 stained positive for CK7 which occurs in a rare subset of patients with CRC, and is often associated with a poor prognosis [20]. For the three most extensively studied PDTOs in this study, immune staining of epithelial CRC markers shows comparable patterns (Supplementary Fig. S1). Furthermore, genomic sequencing revealed individual mutations that are typical for CRC, as well as overall tumour mutational burden values that are conform with MSS CRCs [21] (Fig. 1B). Of the 5 PDTOs, three are *KRAS* mutant, specifically *KRAS G12A* (PDTO013 & PDTO024) and *KRAS G12V* (PDTO026); PDTO018 is *RAS* wild-type and PDTO009 is *BRAF V600E* mutated—in accordance with available patient data (Fig. 1B & Supplementary Table S1). Overall, the PDTOs retain the histopathological features and genotypic architecture of the patient’s tumours, and present reasonable mutational diversity including *RAS* status.

### PDTOs are sensitive to multikinase TKIs

We next selected TKIs with promising physiochemical and pharmacokinetic properties towards intermittent high-dose therapy. Besides regarding the Lipinski Rule of 5 and other parameters for bioavailability, we mainly selected for multitarget inhibitors with increased potential at slightly higher concentrations levels [14] (see methods and Supplementary Table 2). This resulted in sunitinib (mainly targeting PDGFRs, VEGFRs, KIT, FLT3, CSF-1R, RET), cediranib (PDGFRs, VEGFRs, FGFRs), and osimertinib (EGFR T790M mutant, HER2,4, ACK1, BLK) as top candidates [22–24]. To contrast these multikinase inhibitors with a TKI that is more selective and has lower off-target potential yet has similar pharmacokinetic and physiochemical properties, we chose imatinib (Bcr-Abl, CSF/c-kit).

To determine the sensitivity of 3D matrix-embedded PDTOs towards these TKIs, we performed an exposure-response analysis and calculated IC_50_ values, adding the drugs to the culture medium surrounding the 3D matrix-embedded PDTOs (Supplementary Fig. S2A). Sunitinib had the highest IC_50_ at 2.1–10.4 µM medium concentration (MC), followed by cediranib (2.6–7.1 µM MC) and osimertinib with the lowest IC_50_ (1.4–3.8 µM MC) (Supplementary Fig. S2B–C). These results show that continuous treatment (168 h) with osimertinib at MCs of 7.5 µM is sufficient to inhibit growth in all the PDTOs, whereas 15 µM MC is needed for cediranib and sunitinib.

### HDST TKI treatment successfully inhibits the growth of mCRC organoids

**Fig. 2:**
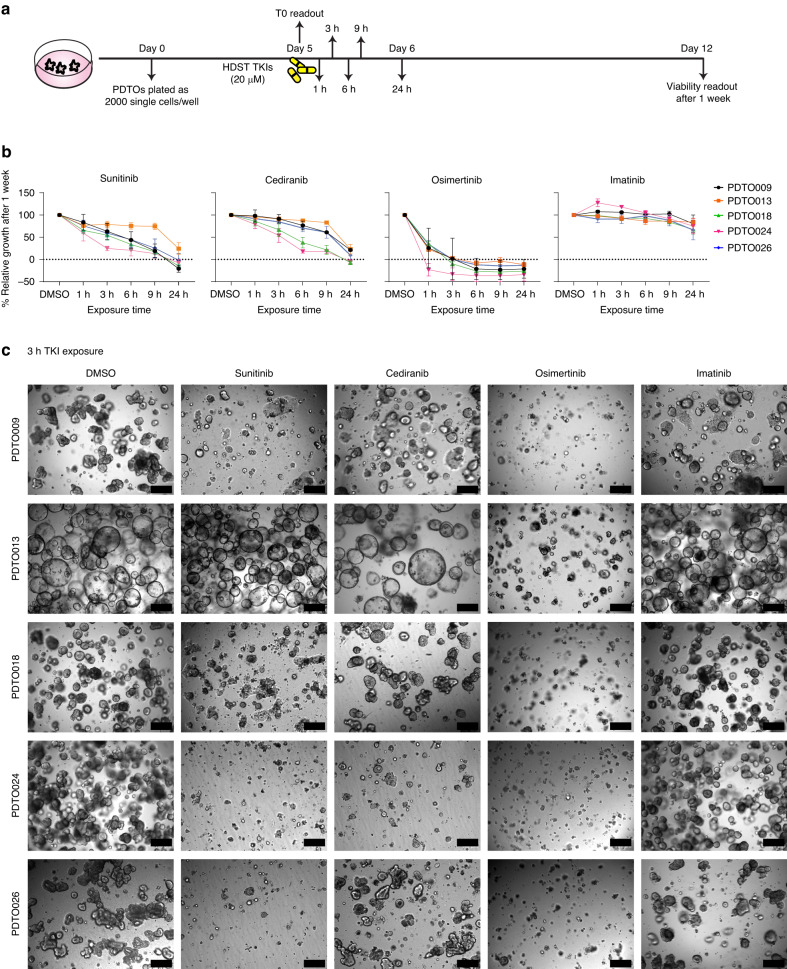
High-dose, short-term TKI treatment on mCRC PDTOs. **A** Schematic representation of the timeline of HDST TKI exposure on mCRC PDTOs. The PDTOs are grown from single cells for 5 days and then exposed to 20 µM MC of selected TKIs for different time intervals (1–24 h) on day 0. After wash-out, the PDTOs are further grown in fresh drug-free medium until cell viability is measured on day 7. **B** PDTO growth on day 7 after HDST treatment. Relative growth is normalised to DMSO control (100% growth) and a t_0_ measurement (0%). All experiments were performed in triplicate and are presented as averages from 3 independent biological replicates with standard error of the mean. **C** Brightfield images showing the effect of 3 h HDST TKI treatment, taken on day 7. This depicts a clear change in morphology compared to DMSO indicating a strong treatment effect HDST TKI exposure. Scale bar = 200 µm. HDST High-dose short-term, TKIs Tyrosine kinase inhibitors, PDTOs patient-derived tumour organoids.

To investigate the effect of HDST TKI exposure, we subjected PDTOs to 5 µM, 10 µM and 20 µM MC inhibitor for a short period (1–24 h), followed by drug wash-out and viability measurement 7 days after the start of treatment (Fig. 2A). Comparing overall growth relative to the DMSO control and a t_0_ measurement, the highest growth reduction in all PDTOs was observed using 20 µM MC HDST exposure for all TKIs, although osimertinib was also highly effective at lower medium concentrations (Fig. 2B, Supplementary Fig. S3A–C). For sunitinib 20 µM MC, 24 h exposure reduced growth completely in 4 out of 5 PDTOs and by 75% in PDTO013. For cediranib, the 20 µM MC results were comparable to those for HDST sunitinib, although only 2 PDTOs reached full growth inhibition after 24 h of exposure—the others ranged between 75–90%. Strikingly, an exposure for 3 h to 20 µM MC osimertinib was enough to fully inhibit growth in all PDTOs, including PDTO013 that is somewhat resistant to both sunitinib and cediranib. In contrast, 20 µM MC HDST exposure with the more selective TKI imatinib did not elicit any inhibition in growth of the PDTOs (Fig. 2B). The strong efficacy of HDST osimertinib exposure on blocking PDTO growth is also emphasised by the brightfield images of the 3 h treatment, taken just before the viability readout at day 7 (Fig. 2C).

### HDST TKI treatment induces marked intracellular drug accumulation

Besides predicting anti-cancer efficacy, we also aimed to use our mCRC PDTOs to assess intra-tumoroid drug concentrations associated with drug response. Because intracellular drug concentrations can differ substantially from culture medium concentrations, we developed and validated bioanalytical methods for quantifying drug concentrations in culture medium and disaggregated cell pellets from organoids. First, we assessed the reliability of our organoid-adapted preclinical pharmacology collection protocol by measuring the starting medium concentrations and performing a mass-balance study, in which 99% of the total sunitinib content could be tracked across each step of the process (Supplementary Fig. S4A–E). The starting concentrations were confirmed to be ~20 µM for all experiments (Supplementary Fig. S4A). Interestingly, the culture medium was depleted of 19% of sunitinib (down to ~16 µM; 81%) after just 3 h of exposure to 3D-matrix embedded PDTOs (Supplementary Fig. S4C). While some of the drug could be traced to the removed culture matrix and washing steps, a large fraction was found in the disaggregated single cell pellet of the organoids (Supplementary Fig. S4B, C). We determined intra-tumoroid concentration (ITC) by inferring total cell volume from pellet weight (Supplementary Fig. S4D). Remarkably, the organoids had accumulated 7% of the total sunitinib mass added to the culture medium, amounting to an average ITC of 503 µM (SEM 70); a 25-fold enrichment compared to the starting levels in the medium (Supplementary Fig. S4E).

**Fig. 3:**
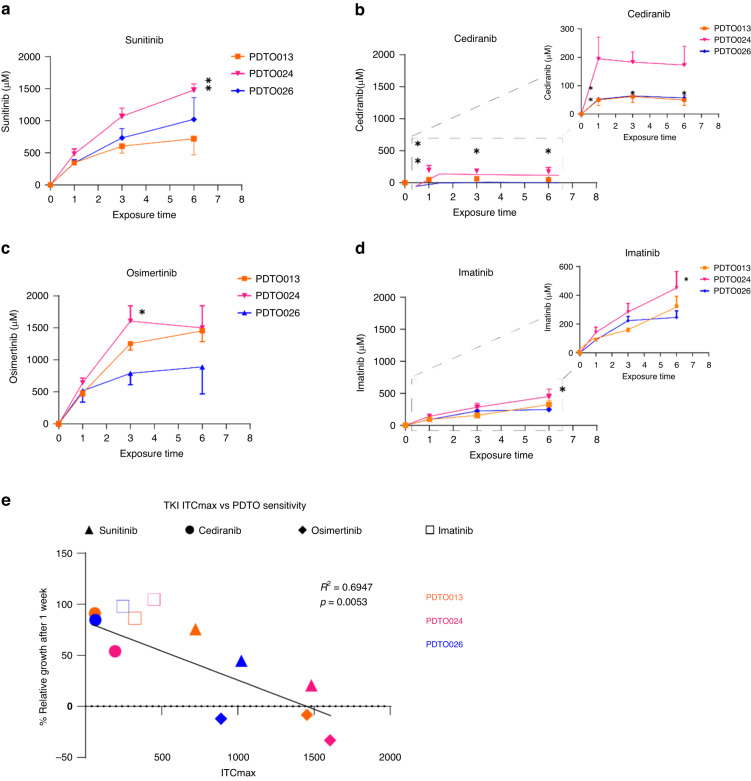
Intra-tumoroid TKI concentrations of the PDTOs and its association with sensitivity towards HDST. **A** The measured intra-tumoroid sunitinib concentrations in PDTOs of varying sensitivities after being exposed to 20 µM sunitinib for 1 h, 3 h and 6 h. **B** Cediranib, **C** osimertinib and **D** imatinib. **E** Association between the highest intra-tumoroid TKI concentration (ITC_max_) versus the percentage of relative growth one week after exposure to 20 µM TKIs for the corresponding exposure times. *P < 0.05, **P < 0.005. Two-way ANOVA test used to determine the difference in the intra-tumoroid TKI concentrations between different PDTOs across different time points. Pearson correlation test used to determine the correlation between the ITC_max_ of multikinase inhibitors (sunitinib, cediranib and osimertinib) vs. % relative growth after a week.

We next measured ITC for all four TKIs over the first few hours of high-dose treatment. Based on HDST treatment responses, we noted that PDTO024 was the most sensitive to all multitarget TKIs and PDTO013 the least sensitive. We included a third *KRAS*-mutant organoid, PDTO026, that showed intermediate sensitivity for the latter TKIs. ITC accumulated steeply over the first few hours for all TKIs, yet—except for cediranib—the ITC still increased somewhat between 3 and 6 h (Fig. 3A–D) and (Supplementary Fig. S4F–H). Reflecting what we saw in the initial mass balance experiment, the maximum values that we measured (ITC_max_) exceeded the starting medium concentration by up to 75–80 fold, for osimertinib and sunitinib, and 3–25 fold for the other TKIs (Supplementary Fig. S4F, G). We conclude that our selection of TKIs with favourable properties for attaining elevated local concentrations yields inhibitors that are indeed capable of accumulation inside cancer cells.

### HDST treatment sensitivity correlates with intra-tumoroid TKI concentration

Interestingly, we observed a significantly higher ITC in the sensitive PDTO024 as compared to the least sensitive PDTO013 (1482 µM *vs* 722 µM; *P* = 0.0030) for sunitinib (Fig. 3A and Supplementary Fig. S4G). A similar difference was observed for cediranib: PDTO024 had a significantly higher ITC than PDTO013 and also compared to PDTO026 (195 µM vs 50.2 µM; *P* = 0.0138, and 195 µM *vs* 52.2 µM; *P* = 0.0153, respectively) (Fig. 3B). Osimertinib, to which all three PDTOs are highly sensitive, accumulated to comparable levels in both PDTO013 and PDTO024, but was significantly lower in PDTO026 ([1453 µM, 1605 µM, and 890 µM, respectively], Fig. 3C). Nevertheless, even the latter ITC represents a ~40-fold enrichment over starting medium concentration. Overall, for all three multitarget TKIs taken together, we noted a significant negative correlation between PDTO growth and ITC_max_ (R^2^ = 0.6947, *P* = 0.0053; Fig. 3E). In contrast, the more selective TKI imatinib, that had negligible activity in HDST treatment, nevertheless showed drug accumulation in the tumoroids (10–20 fold higher than the starting MC), hence giving no indication of exhibiting this correlation (Fig. 3D–E). Taken together, we conclude that the activity of multitarget TKIs in HDST treatment of PDTOs is an exposure-dependent phenomenon that involves high intracellular drug accumulation. Moreover, of the three TKIs selected, osimertinib most effectively inhibits mCRC cell growth.

### HDST osimertinib induces apoptotic cell death in PDTOs more effectively as compared to other TKIs

**Fig. 4:**
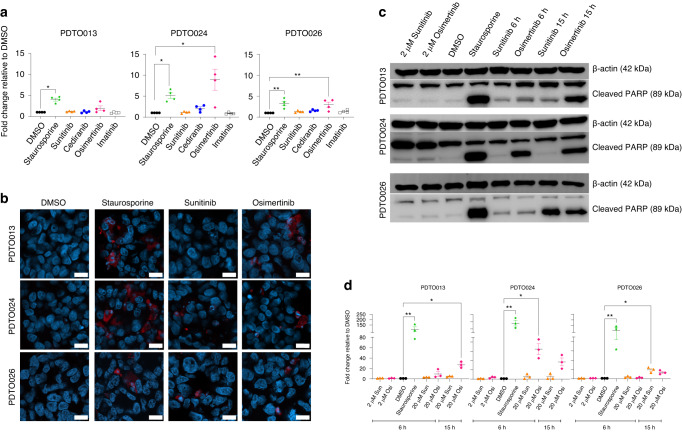
Caspase-3/7 activity, cleaved caspase-3 staining and cleaved PARP after HDST TKI exposure. **A** Enzymatic caspase-3/7 activity measured in PDTO013, PDTO024 and PDTO026 after 3 h of exposure to 20 µM sunitinib, cediranib, osimertinib and imatinib. Ten µM staurosporine is used as positive control and DMSO as negative control. *P < 0.05, **P < 0.005. Kruskal–Wallis test used to determine the difference between treatment conditions. All the experiments were performed with three replicates and repeated at least four times. **B** Immunofluorescence staining of cleaved caspase-3 (red), supported by nuclear DAPI staining (blue), of PDTOs treated with 3 h high-dose sunitinib and osimertinib, 10 µM staurosporine (positive control) or DMSO (negative control) Scale bar = 200 µm. **C** Western blots depicting the expression of cleaved PARP (89 kDa) and loading control β-actin (42 kDa) in PDTO013, PDTO024 and PDTO026 after 6 h and 15 h of 20 µM sunitinib and osimertinib; representative for 3 independent experiments. **D** Quantification of the protein expression depicted in the western blots, n = 3. Values were corrected for loading control b-actin and subsequently normalised for the DMSO negative control. *P < 0.05, **P < 0.005, Kruskal–Wallis test. Experiment was performed as three separate biological replicates.

Besides blocking cancer cell growth, the negative values in Fig. 2B, i.e. fewer cells in treated wells at day 7 than untreated at day 0, indicate that very short treatment with high-dose osimertinib induces cell death. Moreover, the morphological assessment in Fig. 2C supports cell death, as indicated by the presence of dark, fragmented organoid remnants. In fact, images taken just 21 h after 3 h HDST TKI exposure confirms the highly cytotoxic activity of the treatment and suggests apoptotic cell debris (Supplementary Fig. S5A). To assess and quantify regulated cell death, we used a caspase-3/7 enzymatic activity assay at 3 h of treatment with 20 µM MC TKI. This short term high-dose osimertinib exposure already induced a significantly increased caspase-3/7 activity in PDTO024 and PDTO026 (*P* = 0.0183 and *P* = 0.0056, respectively). This trend is also observed for PDTO013 (Fig. 4A). The activation of cleaved caspase-3 after 3 h of HDST osimertinib treatment was confirmed with immunofluorescence staining (Fig. 4B) and its quantification (Supplementary Fig. S5B). Furthermore, concurring with the data in Fig. 2, the other TKIs did not (yet) induce caspase-3/7-mediated cell death at this early time point, although we did observe a slight increase in signal for cediranib in the more sensitive PDTO024. Additionally, an evident increase in cleaved Poly (ADP-ribose) polymerase 1 (PARP), a substrate of caspase-3/7 apoptotic activity, was observed after HDST osimertinib treatment of 6 h and 15 h respectively in all the PDTOs (Fig. 4C). For PDTO013 and PDTO26, the highest PARP cleavage activity was seen at 15 h of HDST, with 27-fold (*P* = 0.023) and 14-fold (*P* = 0.14) increases relative to control (DMSO), respectively. For PDTO024, PARP cleavage was markedly increased at 6 h already (63-fold, *P* = 0.0463, Fig. 4D). No biochemical evidence for apoptosis was observed when the PDTOs were treated with a 10 – fold lower dose of 2 µM osimertinib. HDST sunitinib treatment for 15 h only resulted in a statistically significant increase in cleaved PARP activity in PDTO026 (19-fold, *P* = 0.046). Thus, the high efficacy of HDST osimertinib in our data is substantiated by effective induction of apoptotic cell death in PDTOs.

## Discussion

Intermittent high-dose targeted therapy is based on the concept that drug concentrations responsible for preclinical efficacy—concentrations that exceed levels needed for on-label target inhibition—are too often not reached inside tumours by standard dosing in patients [14]. So far, the mechanistic consequences of such elevated intra-tumoral drug levels—especially with TKIs of which many are not very selective—have been poorly explored. In this study, we demonstrate preclinical efficacy of HDST-treated multitarget TKIs against mCRC PDTOs, and directly link efficacy to pronounced local drug accumulation that leads to apoptotic cell death a few hours after treatment start. These results increase our understanding of the mechanism-of-action of this strategy and, together with supportive early clinical evidence, may aid in bringing improved benefit from TKI treatment to patients with advanced CRC.

The success rate for TKIs that show initial promise in preclinical studies (against CRC) is poor. One crucial factor governing the translational gap is the lack of predictive complex preclinical models. Cell lines remain a widely used model to test TKIs in mCRC [12]. Due to their inability to reflect mCRC heterogeneity, plastic-adapted (2D) cell lines have serious limitations [25, 26]. Alternatively, 3D extracellular matrix-encapsulated PDTOs have demonstrated an ability to maintain genetic heterogeneity as well as the phenotypic architecture of the patient tumour, and therefore are considered more relevant to study alternative treatment strategies and pharmacology [26, 27]. Recent work has shown great potential of PDTOs as models to predict and study therapy resistance [28, 29]. Although organoids are increasingly used in both fundamental and translational research, their potential as the preferred modality for (pre-) clinical efficacy and pharmacological research remains only partially explored. These models could be further improved with the addition of components from the tumour microenvironment (TME) to study drug distribution, but also because the TME can be a significant determinant in therapeutic response [17, 30] or can harbour relevant targets itself [12, 31, 32].

Another barrier is the challenge of translating preclinical drug concentrations to patient dosing; to determine and implement the required exposure for efficacy in the clinic. Indeed, as sparse as reliable clinical data on intra-tumoral drug levels are, preclinical studies rarely assess effective intracellular drug exposure [12]. Therefore, our pharmacological evaluations in TKI-treated PDTOs are pertinent in providing unprecedented insight in the relation between activity and resistance based on intra-cellular drug concentrations. Our data reveal that intra-tumoroid TKI concentrations can differ substantially from medium concentrations. Reasons for this may be drug metabolism, active transport across cell membranes, or pH-dependent sequestration [33]. Indeed, sunitinib has been shown to be retained in lysosomes after protonation [34, 35]. In fact, all four selected TKIs are weak bases with multiple amines as proton acceptors, which may account for the marked intra-tumoroid accumulation we observed. Nevertheless, there may be additional (combined) mechanism that could explain individual levels of sensitivity or resistance—and their future elucidation may be facilitated by PDTOs. Furthermore, the use of stromal cells and healthy tissue organoids can help to determine whether this is tumour or cell type-specific, and thereby contributes to predict patient responses [36]. Nevertheless, more complex models (e.g. 3D co-cultures, *ex vivo* tumour explants, microfluidic devices) will be needed in future work to study true drug dynamics at clinically relevant exposure levels.

The concept of marked tumoral TKI accumulation—compared to plasma levels—agrees with prior observations in several clinical studies, especially in high-dose strategies [16, 37, 38]. Intermittent high-dose sunitinib therapy has shown safety and feasibility in a heterogeneous group of patients with advanced cancers including mCRC [15]. Interestingly, similar to our sunitinib ITC:MC ratio of approximately 25-fold in PDTOs, intra-tumoral sunitinib concentrations was found to be 10–88-fold higher than plasma levels [16]. Moreover, within that range, the preliminary data suggest potential PFS and OS benefit in correlation with the level of intra-tumoral drug accumulation [16]. The mechanistic implications of widening the concentration-dependent spectrum of kinase inhibition of TKIs in order to boost anti-cancer drug activity are poorly defined, but may encompass simultaneous targeting of disparate oncogenic pathways. This would likely involve moderate- or even low-affinity targets—of which many TKIs have multiple. In fact, HDST–TKI efficacy may be to a large extent independent from on-labels targets such as a mutated EGFR in the case of osimertinib [22–24]. Although in vitro binding or kinase assays can give precise data on drug affinities, the actual range of targets and pathways that are inhibited in tumours or PDTOs needs to be identified empirically in order to better understand the mechanism. Proteomics in both PDTOs and patient samples, as well as cellular binding assays, may help unravel the presumably complex combination of inhibited processes, and perhaps improve selection criteria for multitarget TKIs based on their selectivity profile [13, 14].

Differential TKI effectiveness in HDST treatment in our study likely stems from the interplay between selectivity and the capacity for intra-tumoroid accumulation. The most anti-proliferative drugs we found were osimertinib and sunitinib, both TKIs with many ‘off-targets’ that reached remarkably high intra-tumoroid drug concentrations in a short time-frame. Of the other two TKIs that showed somewhat less striking intracellular accumulation, the multitarget TKI cediranib showed considerably strong activity, whereas imatinib, a much more selective drug [39–41], did not. The fact that osimertinib is the only TKI in our selection that can covalently bind to its targets may partially explain its superior efficacy in conditions of drug excess [22]. Indeed, it may be the reason for the absent relationship between intra-tumoroid drug accumulation and PDTO growth; indicating a potential cytotoxic threshold that is reached in a short time period in all three PDTOs. Nevertheless, the difference may also be related to the kinome selectivity profile. Of note, although osimertinib is sometimes considered to be selective, it actually has multiple off-targets at a slightly higher concentration [22]. Furthermore, there is supportive evidence linking osimertinib to apoptosis in non-small cell lung cancer (NSCLC) cells, even at the low in vitro medium concentrations of 0.1–4 μM [42, 43], and there have been clinical studies doubling the daily dose for patients with advanced NSCLC reported modest benefits [44, 45]. The safety and feasibility of substantially higher (intermittent) osimertinib dosing remains to be clinically tested, which we argue should incorporate on-treatment biopsies for pharmacological assessment.

Given the correlation between intra-tumoroid drug concentration and efficacy, the variability between PDTOs in the former may give insight into levels of individual sensitivity or resistance. For sunitinib and cediranib, this difference in accumulated concentration may be related to the expression of drug efflux transporters, including the P-glycoprotein pump (P-gp). P-gp is known to play a role in multi-drug resistance in CRC [46] and other cancer types [47–50], and higher expression of P-gp is usually correlated with a worse prognosis [51, 52]. Sato et al. have shown that combining P-gp inhibitor elacridar with sunitinib enhances the cytotoxic effect in a renal cell carcinoma model [53]; and it would be interesting to investigate the effect of such a P-gp inhibitor on drug accumulation and sensitivity in our HDST setting, including in TKI-resistant PDTO CRC models. Off note, previous studies showed that osimertinib can inhibit drug efflux transporter pumps itself [54], suggesting protection from this potential resistance mechanism. Furthermore, the role of lysosomal sequestration in drug accumulation, whether a potential mechanism for tumoral sensitisation or for cellular resistance [34, 35], requires further investigation.

*KRAS*-mutant mCRC urgently needs more (targeted) therapeutic options; patients with these cancers do not respond to the otherwise clinically beneficial strategies blocking the pathway KRAS is involved in signalling [5, 55, 56]. Although recent work from Fukada et al. has shown that harbouring a *KRAS-G12V* mutation causes osimertinib resistance in NSCLC [57], we show high drug activity in all three *KRAS*-mutant mCRC PDTOs. Interestingly, PDTO026 carries a *KRAS-G12V* mutation and although it is the least sensitive PDTO in our continuous low dosing experiment, this lack of response appears to be overcome by HDST treatment. This suggests that the compounded inhibitory profile associated with HDST treatment potentially affects parallel oncogenic signalling pathways, or brings about an unrelated, direct cytotoxic combination of inhibited proteins. Both a better understanding and clinical validation of HDST osimertinib therapy is highly relevant for patients with *KRAS*-mutated mCRC.

Our study has its limitations. First, we used only 3–5 PDTOs out of our biobank for this study. One of the drawbacks of using PDTOs is the amount of time and expense associated with the growth and expansion of these models. This is especially true for the preclinical pharmacology experiments developed herein. Given the low number of organoids, representing limited heterogeneity in tumour subtypes, or even *KRAS* mutations, it is indeed difficult to extrapolate our findings to a larger scale. Also, PDTOs cannot capture all tumour characteristics and in that sense do not guarantee to predict individual patient responses. However, by using multiple drugs with differential kinase inhibitory profiles, our results clearly demonstrate the relevance of evaluating drug concentrations in preclinical tumour models to improve our insight in their mechanism of action. Furthermore, seemingly relevant TKIs for mCRC did not meet our selection criteria (methods) and therefore, were not tested. For example, regorafenib, although registered for CRC, dose-proportional pharmacokinetics is limited to low dose levels (EMEA/H/C/002573/0000). Another potentially interesting drug based on the recent data indicating efficacy in mCRC, fruquintinib [58] was not considered when experiments were initiated. Thirdly, this study elucidated only a small aspect of the complex mechanism-of-action of HDST treatment: high-dose osimertinib readily induces apoptotic cell death in cancer cells, yet relevant cellular drug targets remain elusive. Furthermore, HDST sunitinib treatment entails evidence for apoptosis in only one PDTO, despite appearing almost as cytotoxic as osimertinib (Fig. 2C and Supplementary Fig. S4A). Therefore, it might induce other types of (regulated) cell death in the HDST setting. Additionally, we find really high concentrations in vitro that might be difficult to reach in patient tumours. Although it may be simplistic to directly compare these to MC and ITC, respectively, future research should indicate whether and how in vitro exposure can be extrapolated to feasibly attainable drug levels in the clinical setting—informing the design of phase I/II studies [59, 60]. Such efforts would likely include the exploration of alternative ways to boost plasma and potentially tumour concentrations [61].

In conclusion, our data demonstrate impressive efficacy of HDST TKI therapy in PDTOs, associating with high intracellular drug concentrations and thus, likely, additional inhibited targets. Osimertinib emerged as the most promising candidate, as it readily accumulated in high-dose-treated mCRC PDTOs to rapidly surpass a putative cytotoxic threshold and induced cell death via apoptosis. Besides warranting further mechanistic research to closer investigate the potential off-targets at these high concentrations, our work endorses the testing of clinical safety and feasibility of (intermittent) high-dose osimertinib treatment. More generally, our work paves the way to assessing necessary levels of drug exposure in vitro and relating this to a better understanding of clinical efficacy or lack thereof, and improves our insight at the molecular level. Our study contributes to addressing a missing link between cellular oncology and clinical pharmacology.

## Supplementary information

Supplementary information

## Data Availability

The datasets generated and/or analysed during the study are available from corresponding author on reasonable request.
