# Supplementary material for: High-dose short-term osimertinib treatment is effective in patient-derived metastatic colorectal cancer organoids
Source: BJC Rep. 2024 Apr 3;2:29. doi: 10.1038/s44276-024-00042-0 (PMC11523998; doi:10.1038/s44276-024-00042-0)
Supplement: Supplementary file 1 — Supplementary information [file 44276_2024_42_MOESM1_ESM.docx]

**Supplementary Figures**

**Figure S1**


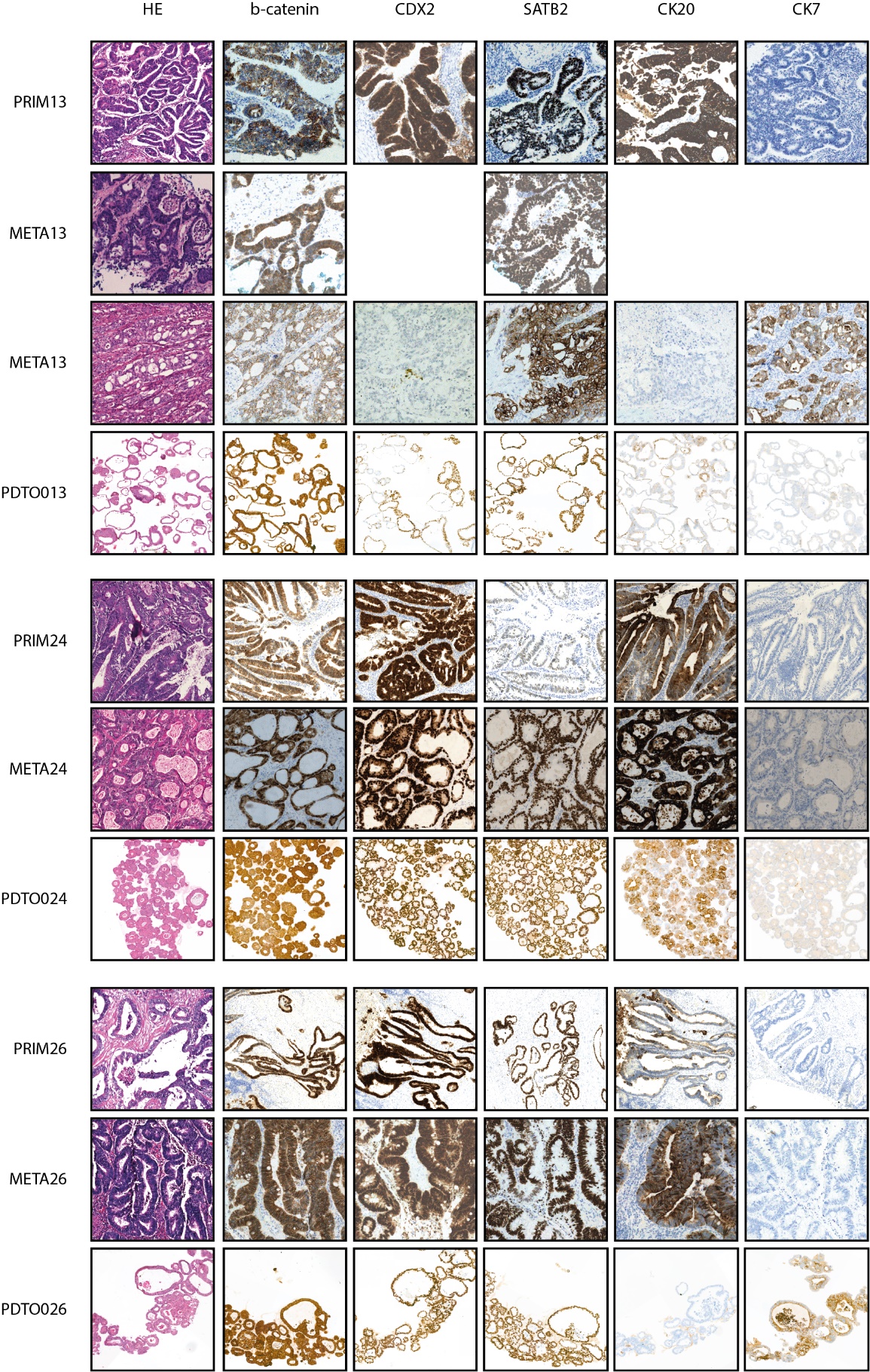


**Figure S1 Histopathological characterization of established mCRC PDTOs and patient tumour.** Histopathological comparison of patient-derived tumour organoids (PDTOs) with metastatic source material and matched primary tumours (where available) by haematoxylin & eosin (H&E) staining, and immunohistochemical staining of 3 extensively investigated mCRC PDTOs with CRC specific markers (β-catenin, CDX2, SATB2 and CK20) and negative marker (CK7).


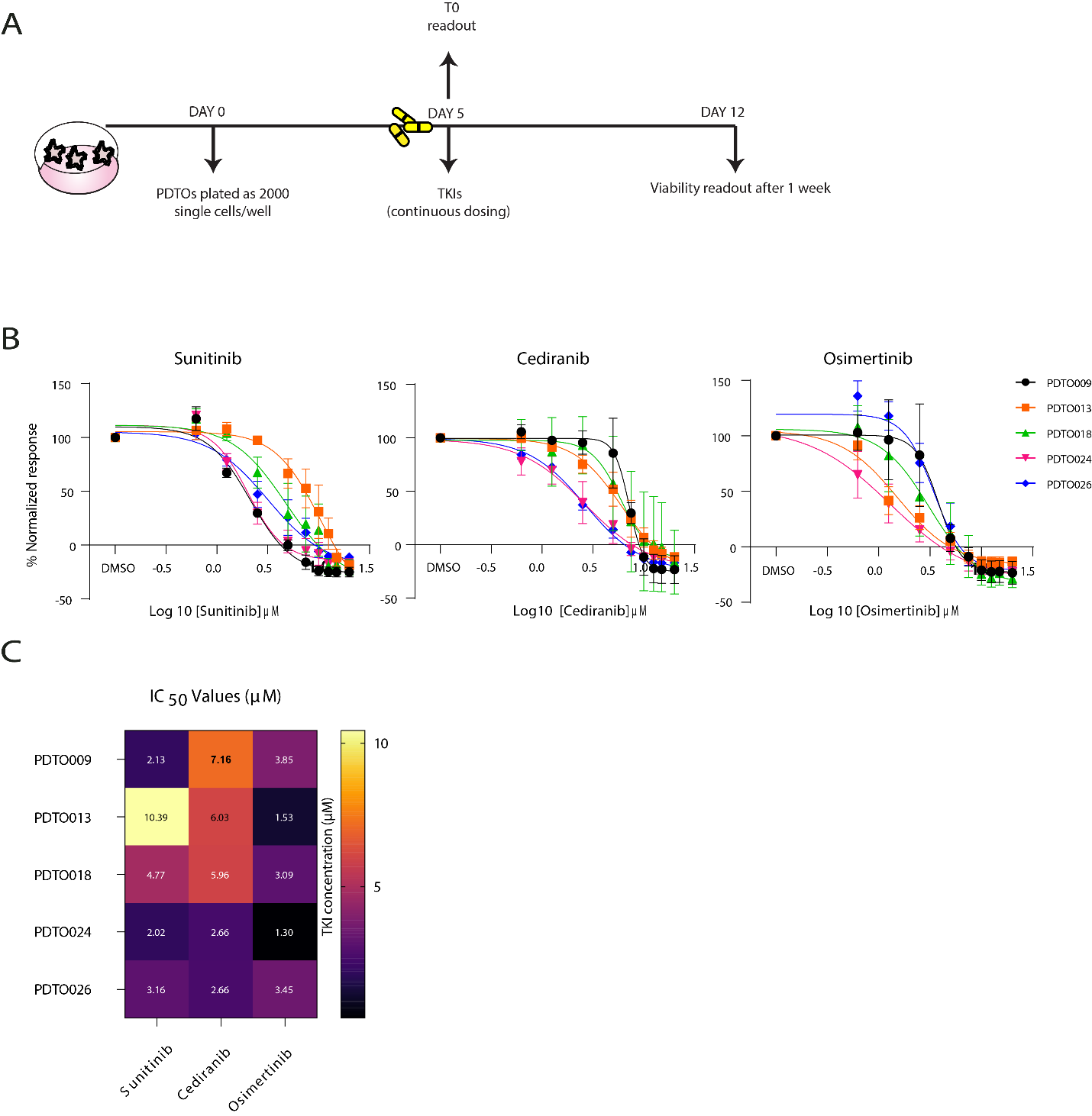
**Figure S2**

**Figure S2 Dose-response analysis of TKI treatment on mCRC PDTOs. (A)** Schematic representation of the timeline of the dose-response analysis of TKIs on mCRC PDTOs. PDTOs are grown from single cells for 5 days and then exposed to different TKI concentrations ranging from 0.6-20 µM for 7 days. Cell viability is measured at day 12. **(B)** Represents the dose-response curves used to determine the IC_50_ values of each TKI in different organoid lines. All experiments were performed in triplicate and are presented as averages from three independent biological replicates with standard error of the mean. **(C)** Heatmap of the IC_50_ values for sunitinib, cediranib and osimertinib for 5 different PDTOs.


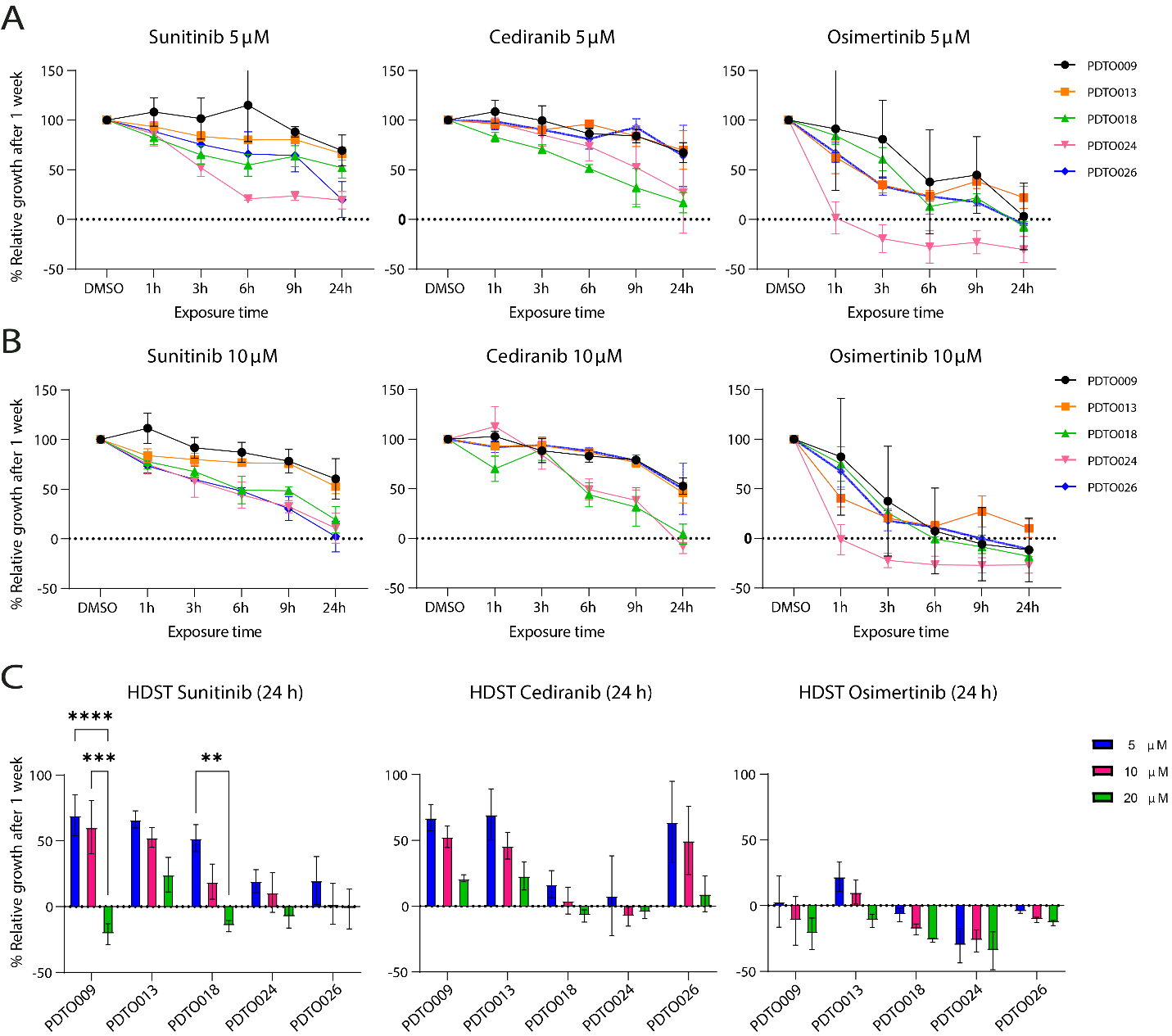
**Figure S3**

**Figure S3 High-dose short-term TKI treatment on mCRC PDTOs. (A)** Represents organoid growth one week after 5 µM, and **(B)** 10 µM HDST TKI treatment. All experiments were performed in triplicate and are presented as averages from three independent biological replicates with standard error of the mean. **(C)** Bar-plots representing a comparison between the percentage growth inhibition after one week of HDST exposure (5, 10 & 20 µM for 24 hours). ** P < 0.005, *** P < 0.0005, **** P < 0.0001, 2way ANOVA Test.


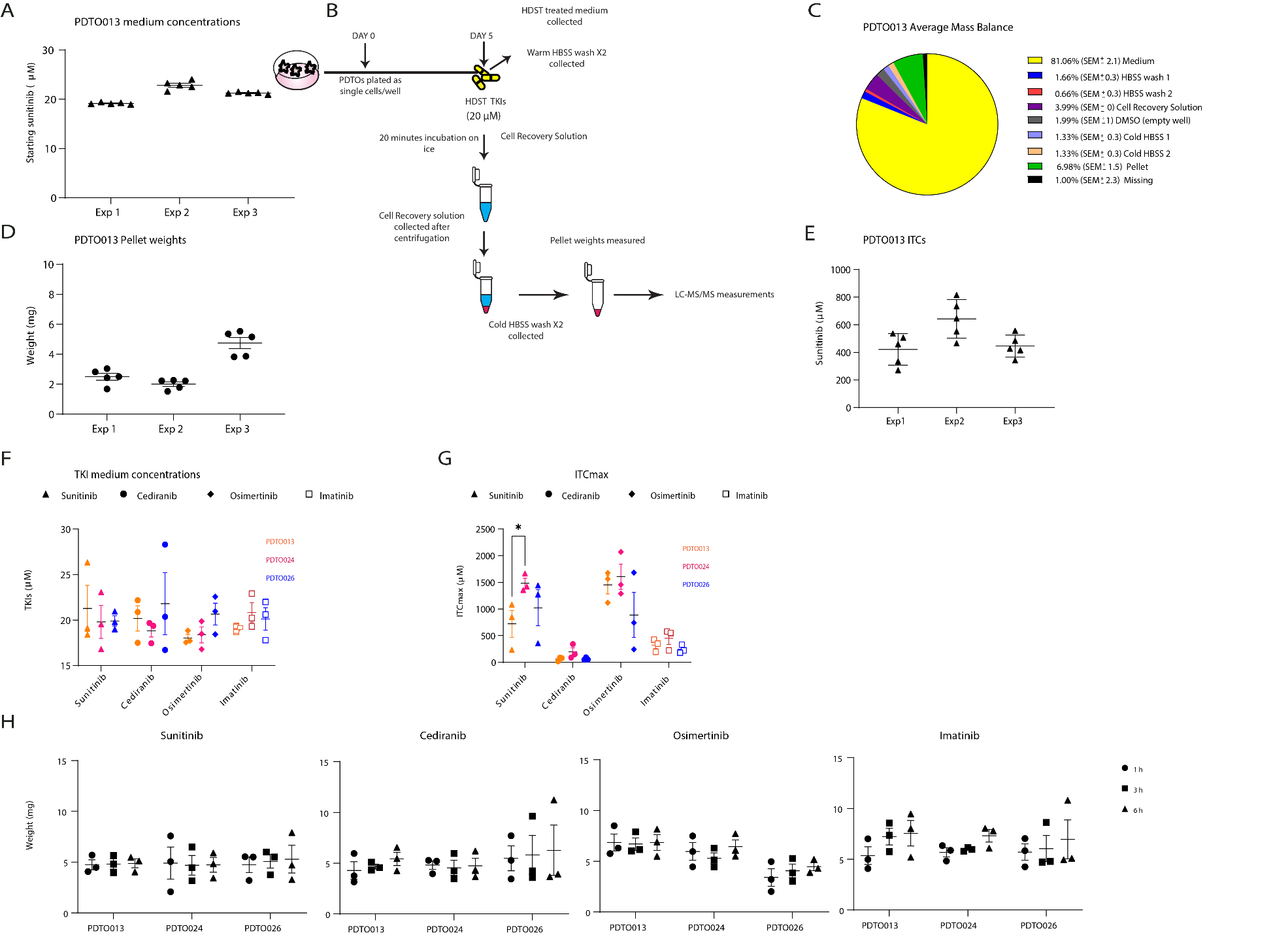
**Figure S4**

**Figure S4 Mass-balance to validate reproducible intra-tumoroid TKI concentration measurements after HDST treatment. (A)** Represents the starting medium concentration of sunitinib used for all the mass-balance experiments to validate intra-tumoroid TKI concentration measurements. **(B)** Schematic representation of the timeline of the mass balance analysis of TKIs on mCRC PDTOs. **(C)** Average percentage of total amount of sunitinib in medium measured before start of exposure (1 ml, 20 µM sunitinib = 7.96 µg) quantified after 3 hours exposure in all steps of the collection process. Data represents the average of three independent biological replicates performed in quintuplicate with the respective standard error of mean (SEM). **(D)** Shows the pellet weights calculated for PDTO013 for the mass-balance experiment after 20 µM HDST treatment for 3 h. **(E)** Average intra-tumoroid sunitinib concentrations after 3 h of HDST-sunitinib treatment in PDTO013. **(F)** Summary of all the starting medium TKI concentrations for each intra-tumoroid concentration experiment. **(G)** Represents the maximum intra-tumoroid concentrations for all the PDTOs treated with HDST sunitinib, cediranib, osimertinib and imatinib. **(H)** Average pellet weights of the PDTOs after HDST TKI treatment for 1, 3 and 6 h respectively for all the four TKIs. Experiments were repeated as three independent biological replicates in duplicate. * P < 0.05, 2way ANOVA test.

**Figure S5**


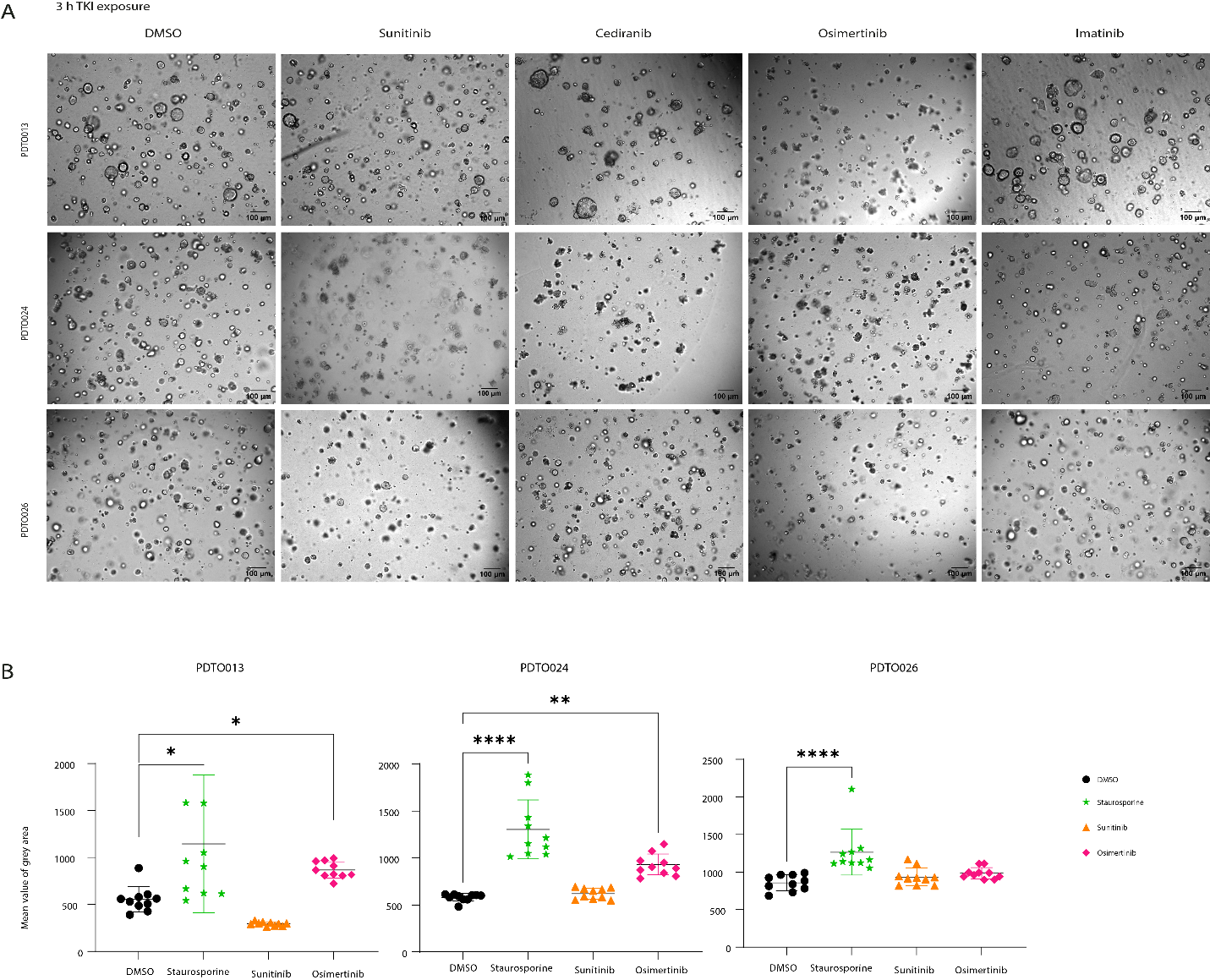


**Figure S5 Morphological cell death and quantification of cleaved caspase-3 IF staining after HDST TKI treatment. (A)** Brightfield images showing the effect of 3 h HDST TKI treatment, taken after 21 hours. This depicts a clear change in morphology compared to DMSO indicating a strong treatment effect HDST TKI exposure. (B) Displays the average of the cleaved caspase-3 channel for each PDTO after HDST TKI treatment. PDTOs were treated with HDST sunitinib and osimertinib, 10 µM staurosporine (positive control) and DMSO (negative). For each condition, ten random images were taken for determining the cleaved caspase-3 activity and the average of all is represented here. * P < 0.05, ** P < 0.005, **** P < 0.0001, Kruskal-Wallis test. Experiment was performed as one biological replicate.

**Supplementary Tables**

**Supplementary Table 1: Patient characteristics**

| **Patient ID** | **Age** | **Sex** | **Primary tumour location** | **biopsy location** | **Sample type** | **Microsatellite instability status** | **Mutational status (BRAF, KRAS, NRAS)** | **Pre-treatment** |
| --- | --- | --- | --- | --- | --- | --- | --- | --- |
| TUM009 | 74 | Female | Left sided colon | Liver | Needle biopsy | MSS | BRAF-V600E | FOLFOX +B  Irinotecan |
| TUM013 | 83 | Male | Right sided colon | Liver | Needle biopsy | MSS | KRAS-G12A | none |
| TUM018 | 64 | Female | Right sided colon | Liver | Needle biopsy | MSS | WT | 4 courses CAPOX |
| TUM024 | 58 | Male | Left sided colon | Liver | Resection | MSS | KRASG-12A | 6 courses FOLFOX |
| TUM026 | 63 | Male | Left sided colon | Liver | Needle biopsy | MSS | KRAS-G12V | none |

**Supplementary Table S1 Patient characteristics.** TUM: Tumour; MSS: Microsatellite stable; WT: Wild type; FOLFOXIRI: Folinic acid, 5-fluorouracil, oxaliplatin and irinotecan; CAPOX: Capecitabine and oxaliplatin; FOLFOX: Folinic acid, fluorouracil and oxaliplatin.

**Supplementary Table 2: TKI properties pertaining to our selection criteria**

| **Properties** | **Sunitinib** | **Cediranib** | **Osimertinib** | **Imatinib** |
| --- | --- | --- | --- | --- |
| **Known targets** | PDGFRα and β,VEGFR1,2,3,KIT, FLT3, CSF-1R, RET | VEGFR1, 2, 3, PDGFRs, FGFRs | EGFR T790M mutant, HER2,4, ACK1, BLK | Bcr-Abl, PDGF, CSF/c-kit |
| **Molecular Weight** | 398.5 (g/mol) | 450.5 (g/mol) | 499.6 (g/mol) | 493.6 (g/mol) |
| **logP** | 2.9-5.2 | 3.77 | 4.47 | 3.47-4.3 |
| **Hydrogen bound donor** | 3 | 1 | 2 | 2 |
| **Hydrogen bound acceptor** | 4 | 7 | 7 | 7 |
| **Lipinski rule of five** | No | Yes | Yes | Yes |
| **pKa** | 8.95 | 9.8 | 9.5; 4.4 | 8.07; 3.73; 2.56; 1.52 |
| **BSC class** | 3 | Not Determined | 3 | 1 |
| **Bioavailability** | Not Determined | Not Determined | >80% | > 95% |
| **Dose proportional PK** | Yes | Yes | Yes | Yes |
| **Protein binding** | ~95% | ~95% | ~99% | ~95% |
| **Plasma elimination half-life** | 40-60 hours | 12-35 hours | 44 hours | 18 hours |

**Supplementary Table S2: Criteria for the selection of the TKIs for High-dose short-term treatment.** 1) An octanol–water partition coefficient (log P) that does not exceed 5, because this will ensure optimal membrane passage; 2) pKA strongest acid >2⋅5 and strongest basic <11, because this will lead to optimal absorption; 3) excellent solubility (BSC class 1 or 3); 4) high bioavailability (or the option to enhance this) and 5) the potency to inhibit multiple “off” target kinases with increased exposure.

**Supplementary Methods**

**Patient-derived tumour organoid culture**

Both needle and resection biopsies were collected on ice in a collection medium constituting of Advanced DMEM/F-12 (GIBCO, 12634010) supplemented with 10 mM HEPES (GIBCO, 15630056), 2 mM GlutaMAX (GIBCO, 35050038), 100 U/mL penicillin/streptomycin (further referred as base medium) with 10 μM Y-27632 (Selleckchem, S1049). Biopsies were mechanically dissociated and collected in base medium. After centrifugation at 200 x g for 3 minutes at 4°C, the pellet was washed with base medium and centrifuged. The pellet was resuspended and further digested at 37°C in base medium supplemented with 5% Noggin conditioned medium, 5% R-Spondin conditioned medium (both in-lab production), 0.2 mg/mL Normocin (InvivoGen, ant-nr-1), 1x N-2 Supplement (GIBCO, 17502048), 1x B-27 (GIBCO, 12587-010), 1,25 mM n-Acetylcysteine (Sigma-Aldrich, A9165), 50 ng/mL human recombinant EGF (Peprotech, AF-100-15-1mg), 10 nM Gastrin-I (Tocris, 3006), 10 mM Nicotinamide (Sigma-Aldrich, N0636), 3 µM SB202190 (Seleckchem, S1077) and 2 µM LY2157299 (in-lab production) further referred as +14 medium with the addition of 20 mg/ml Collagenase from clostridium histolyticum (Sigma-Aldrich, C9407) and 10 μM of Y-27632. A needle biopsy was digested for 30 minutes and resection material up to 2 hours. The tube was vortexed every 5-7 minutes. After tissue digestion, 10% Fetal Calf Serum (FCS; SIGMA, F7524) was added to stop the collagenase digestion. The minced and digested sample was passed through a pre-wetted 200 μM cell strainer (Pluriselect, ITK Diagnostics). After centrifugation, the pellet is carefully resuspended in 70 µl of 70% (diluted with base medium) Reduced Growth Factor Basement Membrane Extract (RGF BME; Type2, PathClear, #3533-005-0) containing 10 µM Y-27632. The cells mixed with BME were seeded as domes in a pre-warmed 24-well plate and incubated upside down at 37°C until the domes solidified. After this +14 medium supplemented with 10 µM Y-27632 was added to the plates. The medium was refreshed twice a week from here on. PDTOs were passaged every week and used for experiments until passage 25.

**Immunohistochemistry (IHC) staining**

To check whether the PDTOs matched the patient tumour phenotypically and to confirm their CRC origins, haematoxylin and eosin (H&E) and CRC specific staining were performed. A CK7 staining was also included in the panel as a negative marker for CRC origins. The PDTOs were fixed in 4% paraformaldehyde (PFA), centrifuged and re-suspended in 2.25% agar (Eurogentec). Both H&E and IHC were performed on the same tissue block. IHC staining was performed with antibodies targeting β-catenin (RTU, DAKO/Agilent); CDX2 (RTU, DAKO/Agilent); SATB2 (diluted 1:80, Cell Marque); CK20 (RTU, DAKO/Agilent) and CK7 (RTU, DAKO/Agilent). These staining were detected using EnVision Systems (DAKO/Agilent).

**Exposure-response analysis**

For each experiment, 2000 single cells were plated in white 96-well microplates with a clear bottom in +14 culture medium. A t_0_ plate was also plated. The single cells were grown for 5 days to form PDTOs. On day 5, the PDTOs were treated with TKIs that were added at 9 different concentrations, ranging from 0.625 µM to 20 µM. The growth rate was determined after 7 days of drug exposure (day 12) by using the CellTiter-Glo® (CTG) 3D Luminescent Cell Viability assay (Promega, G9682) and compared with the t_0_ measurements. Luminescence was measured by a plate reader Victor3^TM^ (PerkinElmer). Percentage DMSO-only treated cells were used as negative control for the assay and the relative growth was compared to this control to determine the half-maximal inhibitory concentration (IC_50_) of the TKIs. All IC_50_ values were calculated using GraphPad Prism (version 9.4.1) using log(inhibitor) *vs* variable slope (four parameters) function. All experiments were performed in triplicate and repeated at least as three biological independent replicates.

**High-dose, short-term exposure of TKIs**

PDTOs were plated as single cells and allowed to grow back into PDTOs for 5 days. On day 5, TKIs (5, 10 and 20 µM) were administered to the PDTOs for various time intervals (1, 3, 6, 9, 24h). After each time interval, the drug containing medium was removed and the wells were washed with pre-warmed HBSS (Lonza, 14175095) and replaced with fresh +14 culture medium. After wash-out, the PDTOs containing the +14 culture medium were allowed to recover until 7 days after treatment with the TKIs, and the medium was refreshed twice in between. On day 12, the readout was performed by CTG assay. PDTO growth was calculated relative to DMSO-treated controls on day 12 (100% growth) and untreated organoids at day 5 (0% growth). All the HDST experiments were performed in triplicate and repeated at least as three biological independent replicates.

**Validating mass balance and determining intra-tumoroid TKI concentrations**

For each mass balance experiment, 100,000 single cells per well were plated in a 24 wells plate and the PDTOs were allowed to grow for 5 days as previously described. On day 5, 20 µM of sunitinib was administered to the PDTOs for 3 hours. Next, the sunitinib containing medium was collected and the PDTOs were washed twice with pre-warmed HBSS to remove surrounding TKI containing medium. Next, 1 ml of cold Cell Recovery Solution (Corning, 354253) was added to each well, the BME domes were mechanically disrupted by pipetting, collected in pre-weighed 1.5 ml Eppendorf tubes and incubated on ice for 20 minutes. The empty wells were washed with 1 ml DMSO which was also collected to recover any residual drug adsorbed on well surfaces. After 20 minutes, the samples were centrifuged at 2000 x g for 5 minutes at room temperature. The Cell Recovery Solution was removed from the tube and was also collected without disturbing the pellet. The pellet was washed twice in 500 μl ice-cold HBSS and centrifuged at the same settings. Supernatant from these washes were collected and the pellet was centrifuged again at the same settings to completely dry the pellet. The dry pellet was weighed. Thereafter, the media (both before and after treatment), washing steps, and tumoroid samples were analysed to quantify sunitinib. The same method was used to quantify intra-tumoroid sunitinib, cediranib, osimertinib and imatinib concentrations in PDTO samples after 20 μM treatment for 1 h, 3 h and 6 h. For these experiments, only the media (before and after treatment) and tumoroid samples were analysed to quantify sunitinib, cediranib, osimertinib and imatinib. For quantification, a validated liquid chromatography-tandem mass spectrometry (LC-MS/MS) assays were used. The LC-MS/MS system consist of an Acquity UPLC® H-class combined with a TQ-S micro detector (Waters®, Milford, USA) with MassLynx software. All the intra-tumoroid concentration experiments were performed in duplicates and repeated as three independent biological replicates.

**Quantification of intra-tumoroid TKI concentrations**

Detection of the analytes took place at the following mass transitions (precursor > collision ion product); sunitinib 399.2 > 283.1; imatinib 494.3 > 394.2; osimertinib 500.1 > 71.9 and cediranib 451.3 > 112.05. Each analytical run included a calibration curve of sunitinib, imatinib, osimertinib and cediranib spiked in plasma over the concentration range of 2–2000 µg/L; 50–50000 µg/L; 50–5000 µg/L and 5–5000 µg/L, respectively.

In each analytical run quality control samples were analysed in duplicate at three different concentrations of 6, 80 and 1500 µg/L for sunitinib; 150, 2000 and 37500 µg/L for imatinib; 120, 775 and 4000µg/L for osimertinib; 15, 500 and 4000 µg/L for cediranib.

The absence of a matrix effect of medium and tumoroids was shown for each analyte during method development and validation. The stable isotopes sunitinib [2H10]; imatinib [13C,2H3]; osimertinib [13C,2H3] and cediranib [2H8] maleate were used as internal standards (IS) to compensate for changes during the whole sample preparation and measurement process on the LC-MS/MS

Analytes were extracted from medium and tumoroids by methanol induced protein precipitation with spiked with IS. Prior protein precipitation samples are diluted 10-20 times to meet the concentration range of the assay. Dilution integrity was shown for each analyte.

The analyte in organoid was dissolved by adding 500 µL DMSO by vigorously mixing and ultrasonication for both 5 minutes. 50 µL organoid extraction or medium was used for analyses to which 200 µL IS spiked precipitation reagents was added after which samples were vigorously mixed and centrifuged for 5 minutes at 18620 g. Supernatant was separated and analysed by injecting 1 µL into the LC-MS/MS system.

The column used for sunitinib, imatinib and osimertinib was an Acquity UPLC® BEH C18 1,7 µm 2,1 x 100 mm (Waters®, Milford, USA) maintained at 50°C. Cediranib uses a Cortecs® UPLC® C18 1.6 µm 2,1 x 50 mm column (Waters®, Milford, USA) maintained at 60°C.

The mobile phase for sunitinib, imatinib and osimertinib consisted of a combination of phase A (2 mM acetate water buffer) and phase B (2 mM acetate methanol buffer) and was delivered in a gradient. phase B was initiated at 25% and held for 1 min, then increased linearly to 50% at 3 min, 90% at 5 min, and held at 90% at 8 min, then back to 25% until the end of the run of 9 min at a flow rate of 0.4 ml/min. After each injection the needle was washed with 1% (v/v) acetonitrile for 5 sec. The mobile phase for cediranib consisted of water with 0.1% (v/v) formic acid (phase A) and acetonitrile with 0,1% (v/v) formic acid (phase B) and also delivered as gradient. Phase B was initiated at 20% for 0.1min, then increased linearly to 40% at 4 min, 90% at 4.1 min and held 90% at 5 min. Then back to 20% until the end of the run of 6 min at a flow rate of 0.8 ml/min. After each injection the needle was washed with 80/20% (v/v) water/methanol for 5sec.

For calculating the concentration of drugs in organoids the following formula was used: The concentration of analyte was determined from LC-MS/MS data by back calculation to the calibration curve. The volume of the organoid pellet was calculated from its weight and the density of water. The sample concentration was then multiplied by the sum of the volume of DMSO and the volume of the organoids, to yield the mass of analyte in the sample. Intra-tumoroid concentration, then, follows from dividing this mass by the organoid volume.

**Caspase-Glo® 3/7 3D assay**

To detect cell death, 6000 single cells were plated in a clear-bottom 96 wells plate and were grown for 5 days. On day 5, the PDTOs were treated with sunitinib, cediranib, osimertinib and imatinib (20 µM). DMSO was used as a negative control and 10 µM staurosporine (TargetMol, T6680) as positive control. The PDTOs were treated for 3 hours. After the treatment, equal volumes of Caspase-Glo® 3/7 reagent (Promega, G8981) were added, incubated at room temperature for 1 hour and then the luminescence was measured using the Victor3^TM^ plate reader. The intensity of luminescence is indicative for the amount of apoptosis and was measured relative to the fold change DMSO control. All the experiments were performed in triplicate and repeated at least four times.

**Immunofluorescent (IF) staining with Cleaved caspase-3 antibody**

The PDTOs were treated for 3 hours with 20 µM of sunitinib, osimertinib and 10 µM staurosporine as positive control. DMSO was used as a negative control for this assay. Next, the PDTOs were washed twice with pre-warmed HBSS and fixed using 4% PFA for 1 hour and paraffin embedded in blocks as described previously. Next, the PDTOs were cut into 5 µM slides. The slides were stained for cleaved caspase-3 antibody (Asp175) (D3E9) Rabbit mAb (Alexa Fluor® 647 Conjugate) (Cell signalling, 9602) according to manufacturer’s protocol and DAPI (Sigma, D9542). The staining was visualized by LSM900 confocal microscope (Zeiss) using 63x magnification. For quantification, (Supplementary Figure S4) the mean value of grey area of the red channel was measured for 10 images for each treatment condition and compared with a Kruskal-Wallis test.

**Western blot**

The PDTOs were treated with 20 µM of sunitinib, osimertinib for 6 h and 15 h. Ten µM of staurosporine treated for 6 h was used as a positive control. DMSO was used as negative control for this assay, along with a 10­—fold lower dose of 2 µM sunitinib and osimertinib for 6 h. Next, the PDTOs were lysed using a 10 X Cell lysis buffer (Cell signalling technology, 9803S) supplemented with phosphatase (Roche, 4906837001) and protease inhibitors (Roche, 11697498001). Further, the lysates were mixed with 2 X Laemelli sample buffer (Biorad, 1610737), supplemented with β-mercaptoethanol (Gibco, 31350010) boiled for 6 minutes at 96°C and stored at -20°C. The lysates were resolved on a 10% running gel followed by transfer to polyvinylidene difluoride membrane (Millipore). The membranes were further blocked with 5% non-fat milk in wash buffer (TBS + 0.1% Tween-20). Next, immunoblotting was performed with primary antibodies, PARP (Cell signalling technology, #9542) and β-actin (Biorad, MCA5775GA) at 4°C overnight. Next day, the blot was incubated for 1 hour with anti-rabbit IgG HRP (Cell Signalling Technology, #7074) and anti-mouse IgG (H+L) (Invitrogen, A11029). Blots were analysed using the chemiluminescence method (LAS4000) and were developed using Super Signal West Femto (Thermo Fisher Scientific, 34094) reagent. Each experiment was repeated at least three times.
